# Antibody-assisted target identification reveals afatinib, an EGFR covalent inhibitor, down-regulating ribonucleotide reductase

**DOI:** 10.18632/oncotarget.25177

**Published:** 2018-04-20

**Authors:** Cheng-Han Yu, Chi-Chi Chou, Hsin-Fang Tu, Wei-Chieh Huang, Ya-Yeh Ho, Kay-Hooi Khoo, Ming-Shyue Lee, Geen-Dong Chang

**Affiliations:** ^1^ Graduate Institute of Biochemical Sciences, College of Life Science, National Taiwan University, Taipei 10617, Taiwan; ^2^ Institute of Biological Chemistry, Academia Sinica, Taipei 11529, Taiwan; ^3^ Department of Biochemistry and Molecular Biology, College of Medicine, National Taiwan University, Taipei 100, Taiwan

**Keywords:** afatinib, target identification, ribonucleotide reductase, covalent drug, gemcitabine

## Abstract

Afatinib, used for the first-line treatment of non-small-cell lung carcinoma (NSCLC) patients with distinct epidermal growth factor receptor (EGFR) mutations, inactivates EGFR by mimicking ATP structure and forming a covalent adduct with EGFR. We developed a method to unravel potential targets of afatinib in NSCLC cells through immunoprecipitation of afatinib-labeling proteins with anti-afatinib antiserum and mass spectrometry analysis. Ribonucleotide reductase (RNR) is one of target proteins of afatinib revealed by this method. Treatment of afatinib at 10-100 nM potently inhibited intracellular RNR activity in an *in vitro* assay using permeabilized PC-9 cells (formerly known as PC-14). PC-9 cells treated with 10 μM afatinib displayed elevated markers of DNA damage. Long-term treatment of therapeutic concentrations of afatinib in PC-9 cells caused significant decrease in protein levels of RNR subunit M2 at 1-10 nM and RNR subunit M1 at 100 nM. EGFR-null Chinese hamster ovary (CHO) cells treated with afatinib also showed similar effects. Afatinib repressed the upregulation of RNR subunit M2 induced by gemcitabine. Covalent modification with afatinib resulting in inhibition and protein downregulation of RNR underscores the therapeutic and off-target effects of afatinib. Afatinib may serve as a lead compound of chemotherapeutic drugs targeting RNR. This method can be widely used in the identification of potential targets of other covalent drugs.

## INTRODUCTION

The epidermal growth factor receptor (EGFR) is one of four members of the ErbB family along with HER2 (ErbB2), HER3 (ErbB3), and HER4 (ErbB4). Functional ErbB receptors are activated by binding to the corresponding ligands, which leads to receptor dimerization and subsequent autophosphorylation or transphosphorylation on certain tyrosine residues, commencing a signaling cascade involved in the regulation of gene expression and many cellular processes [1, 2]. Mutations or overexpression of EGFR is often found in various human cancers, including non-small-cell lung cancer (NSCLC) [3]. Erlotinib and gefitinib are the first-generation EGFR tyrosine kinase inhibitors (TKIs) with high specificity to EGFR [4]. These two drugs bind reversibly to the ATP binding pocket of the catalytic domain and effectively block the downstream signaling initiated from EGFR ligand binding. However, resistance to these drugs occurs frequently in NSCLC patients due to *de novo* EGFR mutations, especially deletions in exon 19 (EGFRdel19) and the exon 21 L858R mutation (EGFR L858R) [5]. Afatinib developed under Boehringer Ingelheim is a covalent inhibitor of ErbB family with IC50 values of 0.5, 14, and 1 nM for EGFR, HER2, HER4 receptor, respectively [5]. Afatinib contains a Michael acceptor group rendering it covalently reactive to a specific cysteine residue within the catalytic cleft (Cys797 in EGFR, Cys805 in HER2, and Cys803 in HER4) and thus preventing the binding of ATP and kinase activation [6, 7]. As afatinib treatment in NSCLC patients significantly improved progression free survival as compared to the standard platinum-based chemotherapy in two pivotal Phase III studies [8, 9], afatinib has been approved in the US in 2014 for the first-line treatment of NSCLC patients who have EGFR mutations that potentially may cause resistance to gefitinib and erlotinib treatment. Erlotinib, gefitinib, and afatinib have also been investigated in the treatment of head and neck cancer [10–12], and afatinib in treating breast cancer [12–14].

Cellular deoxyribonucleoside triphosphates (dNTPs) pool, required for DNA replication and repair, is replenished by both salvage and *de novo* pathways. Ribonucleotide reductase (RNR) catalyzes the rate-limiting step of the *de novo* pathway converting a ribonucleoside diphosphate to the corresponding deoxyribonucleoside diphosphate. Mammalian ribonucleotide reductase consists of catalytic α (RRM1) and free radical-generating β (RRM2) subunits. The enzyme is allosterically regulated through binding of ATP, dATP, TTP or dGTP to the S site and (d)ATP binding to the A site, both in the α subunit [15]. RRM1 and RRM2 are often overexpressed in cancer tissues including lung [16]. In addition, low RRM2 mRNA expression was associated with a significantly higher response rate in patients treated with docetaxel and gemcitabine [17]. Resistance to gemcitabine has been associated with both RRM1 and RRM2 overexpression [18, 19]. Thus, ribonucleotide reductase becomes as an important target for cancer drug development.

During the development of tyrosine kinase inhibitors (TKIs), structure-based drug design, kinome profiling and cellular assays are routinely used to obtain potent and selective compounds against certain tyrosine kinases [20, 21]. Achieving target specificity may be the ultimate aim of drug development but it requires the knowledge of all targets of the drug. Drug-target network analysis estimated that a drug interacts on average with 6.3 targets [22]. Thus, target identification of small-molecule compounds seems to be the bottleneck of drug development [23]. Due to the method limitation in target identification, most TKIs are only examined among the kinase members in the understanding of inhibitor specificity. Most kinase inhibitors might not be as selective as expected because they also target the ATP-binding site of other protein kinases and other ATP-binding proteins may have ATP binding sites indistinguishable from those in protein kinases [24]. In support of this notion, afatinib reversed ABCB1-mediated multidrug resistance in ABCB1-overexpressing ovarian cancer cells by inhibiting the efflux function of ABCB1 [25] and GW8510, a cyclin-dependent kinase inhibitor, inhibited RRM2 expression through promoting its proteasomal degradation [26]. Therefore, close scrutinization of the potential targets of TKIs, especially those already in clinical use, can lead to better understanding of the binding specificity and the resulting therapeutic efficacy. Here, we offer a newly developed method to identify potential target proteins of afatinib. We raised an antiserum against afatinib, and this antiserum can recognize the afatinib-tagged proteins in the cells. Using this method, target identification by specific tagging and antibody detection (TISTA), we found that afatinib covalently bound to RNR, leading to inhibition of RNR activity, downregulation of the RNR protein level, and cell cycle perturbation in PC-9 cells (formerly known as PC-14). Interestingly, afatinib treatment repressed the upregulation of RNR protein level induced by treatment of gemcitabine. Long-term incubation of low-dose afatinib in PC-9 cells and EGFR-null Chinese hamster ovary (CHO) cells also significantly caused downregulation of RNR protein level. Thus, TISTA has been proved to be one powerful method for target identification of covalent drugs such as afatinib in drug repurposing.

## RESULTS

### Production and characterization of an anti-afatinib antiserum

**Figure 1:**
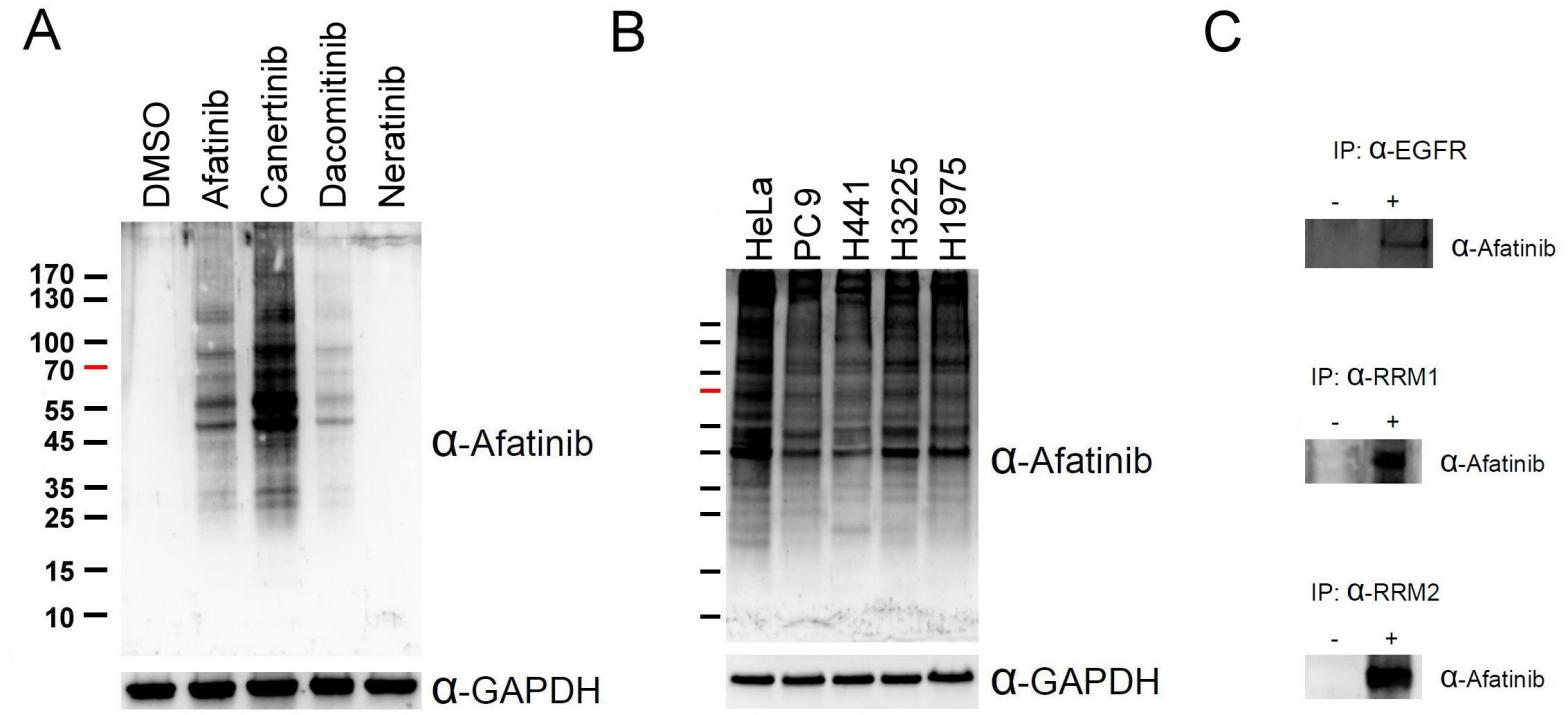
Covalent labeling of cellular proteins in cells by afatinib (**A**) PC-9 cells were treated with 10 μM afatinib, canertinib, dacomitinib, or neratinib in DMEM without FBS for 5 min. (**B**) HeLa cells and four lung cancer cell lines were treated with 10 μM afatinib in DMEM without FBS for 5 min. After drug treatment, the cells were washed with PBS for three times and then lysed with urea lysis buffer containing 1 μM cysteine. The cell lysate was examined by immunoblotting with anti-afatinib antiserum (α-Afatinib) following SDS-PAGE. GAPDH was used as an internal control. (**C**) HeLa cells or PC-9 cells were treated with 10 μM afatinib in DMEM for 1 h and then lysed with IP lysis buffer and then processed for routine immunoprecipitation experiments with anti-EGFR (α-EGFR), anti-RRM1 antibody (α-RRM1) and anti-RRM2 antibody (α-RRM2). The immunoprecipitates were then examined by SDS-PAGE and immunoblotting with anti-afatinib antiserum (α-Afatinib).

Since afatinib was designed as a covalent inhibitor of EGFR [7], we attempted to raise an antiserum against afatinib and use the anti-afatinib antiserum in the identification of afatinib-tagged proteins through immunoprecipitation and LC/MS-MS analyses. The antigen was prepared by coupling of the cysteine thiolate in reduced ovalbumin (OVA) to the alpha carbon of acrylamide group in afatinib under alkaline conditions. We treated PC-9 cells in culture with the various concentrations of afatinib for 1 h and the cell lysate was examined by SDS-PAGE and immunoblotting with the anti-afatinib antiserum (Supplementary Figure 1A). Unexpectedly, numerous proteins were covalently modified by afatinib as showed by the anti-afatinib immunoblotting. However, this antiserum showed high specificity as evidenced by the lack of signal in the control group treated with solvent only and groups treated with low concentrations of afatinib. The signals were readily observed when cells were treated with 1 μM afatinib, and 10 μM afatinib gave rise to higher intensity of signals. Thus, we chose the concentration of 10 μM afatinib in the time-dependent experiments (Supplementary Figure 1B). With increasing incubation time, the intensity of signal increased with an almost identical pattern. In addition, we also performed the afatinib labeling at three pH values. HeLa cells were treated with 10 μM afatinib at pH 6.2, 7.2, and 8.2 for 1 h in culture (Supplementary Figure 1C). The signal of pH 6.2 was weak, and the patterns between pH 7.2 and pH 8.2 were very similar. All tested pH values are lower than the typical pKa of the side chain of cysteine residues. Reactions at a lower pH value appeared to attenuate the afatinib labeling due to the decrease in thiolate formation at the cysteine residues in proteins, confirming the Michael addition mechanism underlying afatinib labeling. Interestingly, the anti-afatinib antiserum can be used to monitor the labeling of other covalent drugs sharing similar structures; canertinib and dacomitinib (Figure 1A). However, the signal of neratinib was undetectable probably due to the lack of the N-chlorofluorophenyl moiety which is present in afatinib, canertinib, and dacomitinib. The higher intensity of canertinib labeling may result from the greater reactivity of canertinib, but not from better recognition by the antiserum. On the other hand, we chose other lung cancer cell lines, H441 (wild-type EGFR), H3225 (L858R EGFR), H1975 (L858R, T790M EGFR), to test whether the mutations in EGFR could influence the afatinib labeling. The results (Figure 1B) showed that there were only slight differences in the afatinib-labeling protein patterns among these four lung cancer cell lines. As EGFR is the known target of afatinib, we attempted to confirm this notion by immunoprecipitation with anti-EGFR antibody followed by immunoblotting with anti-afatinib antiserum using detergent extract from HeLa cells treated with or without 10 μM afatinib for 1 h in culture. As shown in Figure 1C, the data indicate that EGFR can be labeled by afatinib in living cells treated with this drug.

### Ribonucleotide reductase as a novel target protein of afatinib in PC-9 cells

Since many unexpected proteins were ably labeled by afatinib in lung cancer cells, we set up to identify potential targets of afatinib in PC-9 cells using immunoprecipitation and LC/MS-MS analysis. For the positive protein identification, *q*-values were set to 0.01 for both peptides and proteins by controlling the target-decoy strategy to distinguish correct and incorrect identifications. Surprisingly, several deoxyribonucleotide biosynthetic enzymes were found to be potential target proteins of afatinib (highlighted in Supplementary Table 1). RNR received our attention due to its importance as a therapeutic target for cancer drug development. We then used anti-RRM1 antibody or anti-RRM2 antibody to pull down RRM1 or RRM2 from PC-9 cells after afatinib treatment. Indeed, immunoblotting with anti-afatinib antiserum confirmed the tagging of RRM1 or RRM2 with afatinib (Figure 1C). The results showed that RNR was a target protein of afatinib and both subunits formed covalent adducts with afatinib. Thus, the anti-afatinib antiserum is useful for immunoblotting and immunoprecipitation.

**Figure 2:**
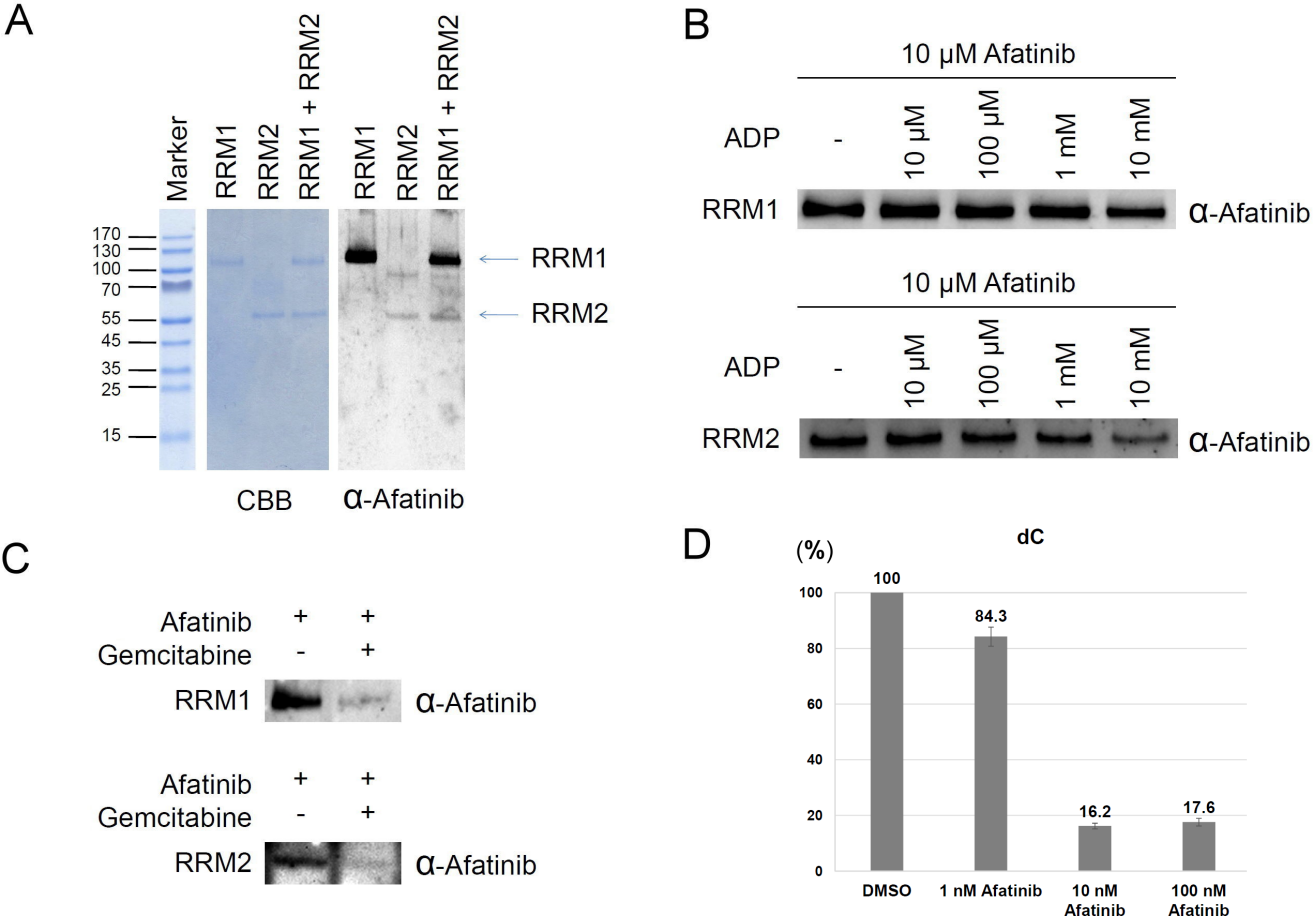
Ribonucleotide reductase as a direct target of afatinib (**A**) The reaction mixture contained 2 μg recombinant RRM1 or/and 1 μg RRM2 in the presence of 12.5 μM afatinib for 1 h at 37° C. The protein concentration was about 0.25 μM. The reaction product was examined by Coomassie blue G-250 staining and immunoblotting with anti-afatinib antiserum (α-Afatinib). (**B**) The reaction mixture initially contained 1 μg recombinant RRM1 or RRM2 in the presence of various concentrations of ADP. After incubation for 15 min, the reaction solution was added with afatinib to a final concentration of 10 μM. The reaction was further incubated at 37° C for 1 h, and then the reaction product was examined by SDS-PAGE and immunoblotting with anti-afatinib antiserum (α-Afatinib). (**C**) The reaction mixture initially contained 1 μg recombinant RRM1 or RRM2 in the presence of 2.5 mM gemcitabine. After incubation for 15 min, the reaction solution was added with afatinib to a final concentration of 10 μM. The reaction was further incubated at 37° C for 30 min, and then the reaction product was examined by SDS-PAGE and immunoblotting with anti-afatinib antiserum (α-Afatinib). (**D**) Rapidly growing PC-9 cells were lysed by freezing and thawing. The cell lysate was treated with afatinib for 1 h and then ribonucleotide reductase activity was measured with the addition of a reagent mixture containing ATP and CDP for 1 h. The reaction product dCDP was extracted from the lysate and treated with alkaline phosphatase. The digested product deoxycytidine was measured with LC-MS analysis.

To determine the modification sites tagged by afatinib on RNR, we incubated recombinant RRM1 or RRM2 protein with afatinib. The reaction product was examined by immunoblotting using anti-afatinib antiserum. As shown in Figure 2A, the results showed that RRM1 protein was apparently modified by afatinib, and RRM2 protein was slightly modified by afatinib. However, RRM2 was modified by afatinib to a more extent when RRM1 and RRM2 were mixed at one to one ratio. After photography, the gel band was excised and processed to determine the modification sites by MS analysis. The amino acid residues at the positions of cysteine 254 and cysteine 492 of RRM1 protein and cysteine 202 of RRM2 protein were identified to be tagged with afatinib (Supplementary Figures 2 and 3).

The three identified sites of RRM1 protein and RRM2 protein were closer to the substrate-binding site in structure than the ATP-binding regulatory site [27], leading to the speculation that afatinib might inhibit the RNR activity via covalent incorporation into the substrate-binding site, thus preventing the entry of substrates. To examine the hypothesis, we performed the *in vitro* afatinib tagging of RNR under ADP competition. RRM1 protein was treated with 0–10 mM ADP first for 15 min, and then the reaction mixture was added with 10 μM afatinib and incubated for an additional 1 h. The results showed that afatinib labeling to RRM1 protein was decreased in the presence of 10 mM ADP (Figure 2B). The same experiment was also performed on RRM2 protein, and afatinib labeling to RRM2 protein was decreased by ADP in a dose-dependent manner (Figure 2B), suggesting that afatinib-binding site in RRM2 is closer to the substrate-binding site. Next, based on the previous studies that gemcitabine was designed to be a RNR inhibitor covalently binding to the substrate-binding site after its conversion to the diphosphate derivative [28] or not [29], we examined whether gemcitabine can compete with afatinib for the substrate-binding site. As expected, pretreatment of 2.5 mM gemcitabine almost completely blocked the afatinib labeling (Figure 2C). In order to directly examine the effects of afatinib labeling on RNR enzyme activity, we established an *in vitro* RNR activity assay using intact cells prepared from rapidly growing PC-9 cells. After membrane disruption by freezing and thawing, permeabilized PC-9 cells in each cultured well were treated with 0–100 nM afatinib for 1 h, and RNR activity was estimated by the amount of dCDP generated following the addition of a reagent mixture containing ATP and CDP for 1 h. Since nucleosides are better resolved and detected than nucleotides in LC-MS analysis [30], the reaction product dCDP was extracted from the reaction solution and treated with alkaline phosphatase. The digested products, deoxycytidine and cytidine, were well separated in LC (Supplementary Figure 4A).Treatment of PC-9 cell lysate *in vitro* with 10 and 100 nM afatinib potently inhibited the production of dCDP (Figure 2D and Supplementary Figure 4B). Thus, these results support the notion that afatinib may directly inhibit RNR activity via covalent occupation of substrate-binding site.

### Decline in RNR protein levels in cells treated with afatinib

**Figure 3:**
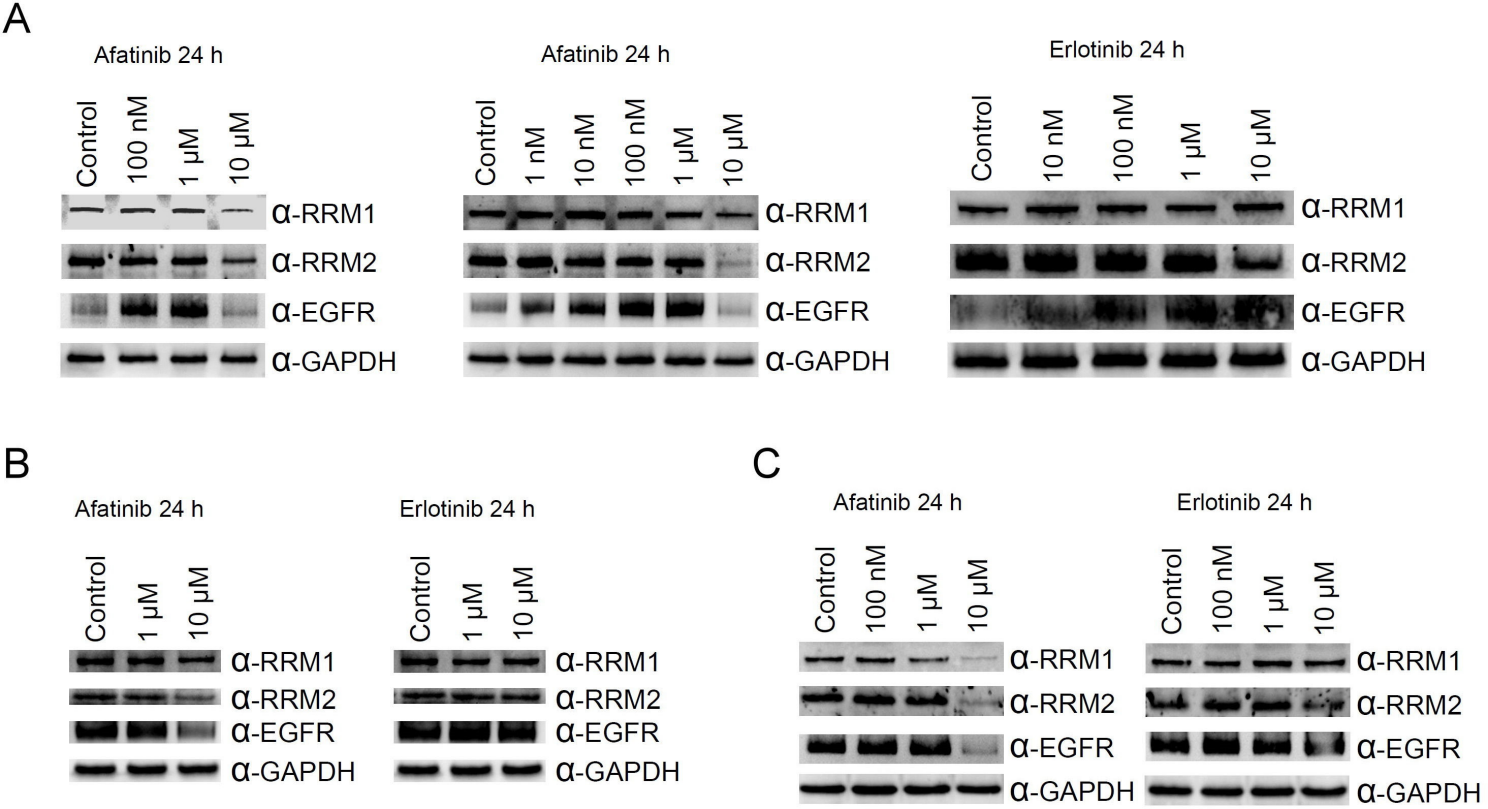
Effects of afatinib and erlotinib on ribonucleotide reductase and EGFR protein levels in different cells PC-9 cells (**A**), NIH3T3 cells (**B**), or COS1 cells (**C**) were treated with afatinib of various concentrations in the absence (middle panel in A) or presence (all others) of FBS for 24 h. After the treatment, the cells were washed with PBS for three times and then lysed with urea lysis buffer containing 1 μM cysteine. The cell lysate was examined by immunoblotting using anti-RRM1 antibody (α-RRM1), anti-RRM2 antibody (α-RRM2), and anti-EGFR antibody (α-EGFR) following SDS-PAGE. GAPDH was used as a loading control.

As RNR was tagged by afatinib, we then examined the protein level of RNR in PC-9 cells with prolonged treatment of afatinib. In cells cultured with FBS, treatment of 10 μM afatinib for 24 h caused decreases in the protein levels of EGFR, RRM1 and RRM2 (left panel, Figure 3A). However, EGFR protein accumulated in cells treated with lower concentrations of afatinib. Similar results were also observed in PC-9 cells cultured in the absence of FBS (middle panel, Figure 3A). In cells treated with 1 μM afatinib, RRM1 levels decreased slightly. We are also curious about whether non-covalent EGFR inhibitors can target RNR. Thus, we chose erlotinib because of its structural similarity to afatinib and performed the same experiments in the presence of FBS. Surprisingly, RRM2 protein level decreased in cells treated with 10 μM erlotinib, and EGFR increased in cells treated with 10 nM to 10 μM erlotinib (right panel, Figure 3A). The same experiments were performed in NIH3T3 and COS1 cell lines, and RRM1, RRM2, and EGFR protein levels decreased in cells treated with of 10 μM afatinib (Figure 3B and 3C). It was worth highlighting that RRM1 slightly declined in NIH3T3 cells treated with 1 and 10 μM erlotinib, EGFR and RRM2 protein levels apparently declined in COS1 cells treated with 10 μM erlotinib. These results demonstrated erlotinib and afatinib target RNR and EGFR resulting in down-regulated protein levels in lung cancer cells as well as in non-cancer cells. Potentially, one non-covalent inhibitor can be derivatized into a covalent analog which then can be used to explore all potential targets by TISTA.

### Induction of DNA damage signal and cell cycle arrest by afatinib treatment

**Figure 4:**
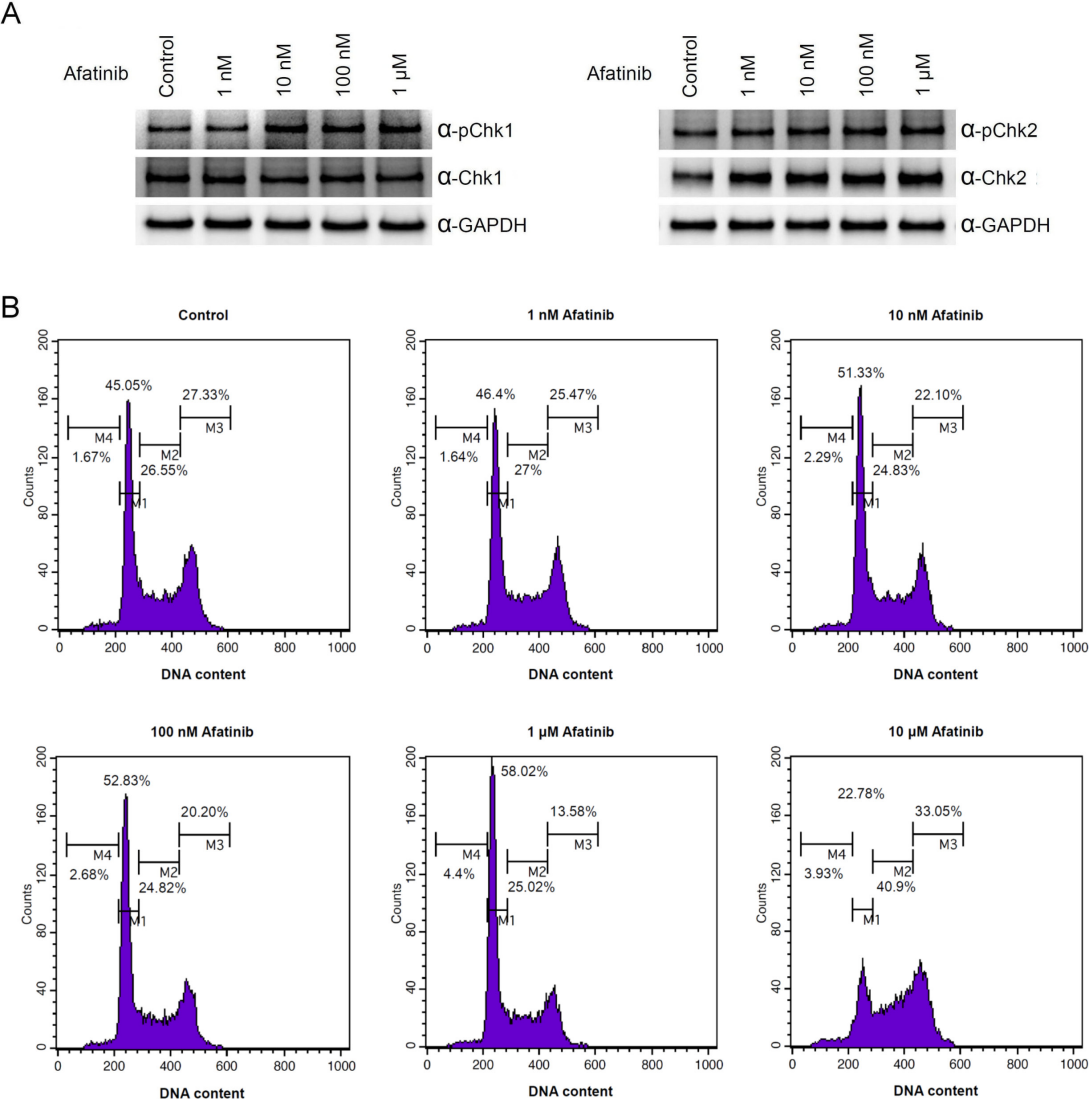
Treatment of afatinib leading to cell cycle perturbation (**A**) PC-9 cells were treated with afatinib in cultured medium containing FBS for 24 h. After the treatment, the cells were washed with PBS for three times and then lysed with urea lysis buffer containing 1 μM cysteine and phosphatase inhibitors. The cell lysate was examined by immunoblotting using anti-Chk1 antibody (α-Chk1), anti-Chk2 antibody (α-Chk2), anti-phospho-Chk1 (Ser 345) (α-pChk1), and anti-phospho-Chk2 (Thr 68) (α-pChk2) following SDS-PAGE. (**B**) PC-9 cells were treated with 1 nM to 10 μM afatinib in cultured medium containing FBS for 24 h. After propidium iodide staining, the cells were subjected to flow cytometry analysis for cell cycle.

Based on the observation that downregulation of RNR protein level occurred after treatment of afatinib in cells, we wondered whether afatinib could cause DNA damage in cells due to the lack of supply of dNTP for DNA synthesis. We found that the signal of phospho-Chk1 increased in cells treated with 10 nM to 10 μM afatinib and the protein level of Chk2 also increased in cells treated with 1 nM to 10 μM afatinib (Figure 4A). These results suggest that DNA damage response and double-strand break occur in cells treated with 10 nM afatinib for 24 h. Cell cycle analysis revealed that treatment of afatinib for 24 h resulted in increases in G1 at 10 nM-1 μM, in sub-G1 at 1–10 μM and in S and G2/M at 10 μM (Figure 4B). As a result, treatment of afatinib at concentrations 10 nM-1 μM inhibited PC-9 cell growth (Supplementary Figure 5). Altogether, afatinib undeniably induced DNA damage response and caused cell cycle arrest possibly via inhibition of DNA synthesis and DNA repair.

### Afatinib down-regulating RRM2 protein level at lower concentrations in long-term treatments

**Figure 5:**
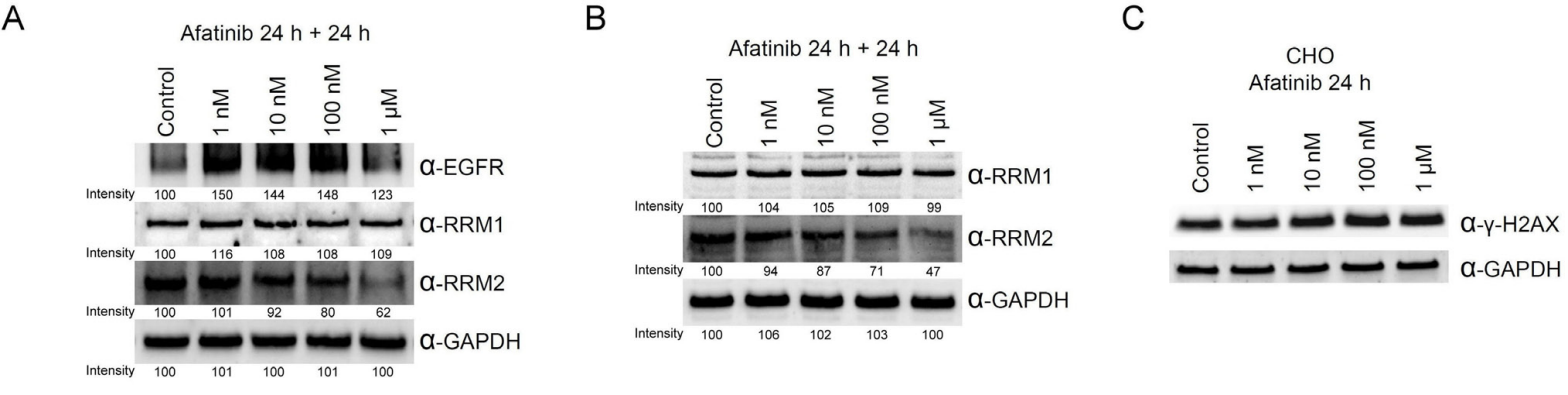
Ribonucleotide reductase is also a target protein of afatinib in PC-9 and EGFR-null CHO cells (**A**) PC-9 cells or (**B**) CHO cells were treated with afatinib in cultured medium in the presence of FBS for 48 h, with a replacement of the culture medium containing afatinib at 24 h. After the treatment, the cells were washed with PBS for three times and then lysed with urea lysis buffer containing 1μM cysteine. The cell lysate was examined by immunoblotting using anti-RRM1 antibody (α-RRM1), anti-RRM2 antibody (α-RRM2), and anti-EGFR antibody (α-EGFR) following SDS-PAGE. (**C**) CHO cells were treated with 10 μM afatinib in cultured medium in the presence of FBS for 24 h, and the cells were immediately washed with TBS for three times and then lysed with urea lysis buffer containing 1 μM cysteine. The cell lysate was examined by immunoblotting using anti-γ-H2AX antibody (α-γ-H2AX) following SDS-PAGE.

**Figure 6:**
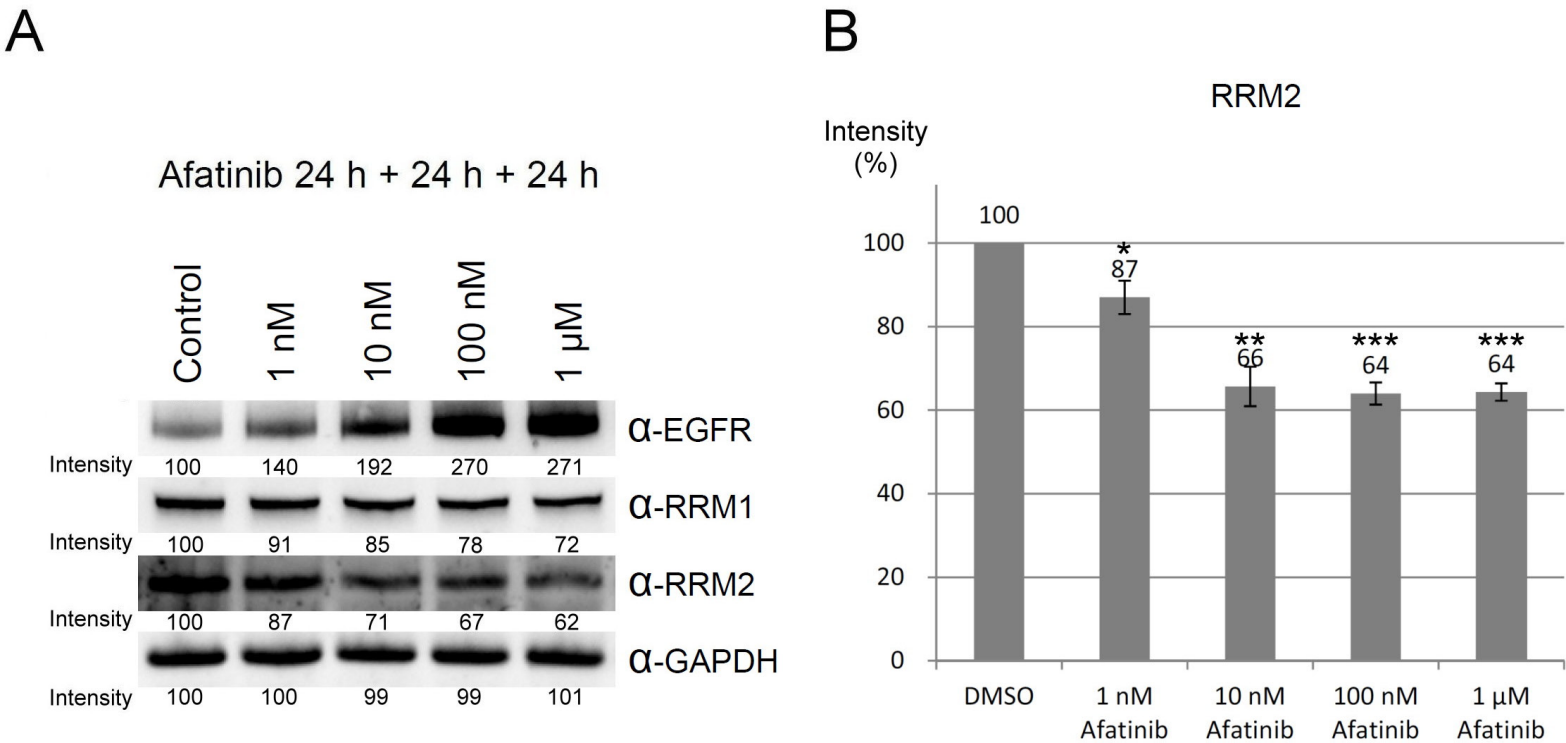
Downregulation of ribonucleotide reductase by long-term treatment of afatinib (**A**) PC-9 cells were treated with afatinib in cultured medium in the presence of FBS for 72 h, with daily replacement of the culture medium containing afatinib. After the treatment, the cells were washed with PBS for three times and then lysed with urea lysis buffer containing 1 μM cysteine. The cell lysate was examined by immunoblotting using anti-RRM1 antibody (α-RRM1), anti-RRM2 antibody (α-RRM2), and anti-EGFR antibody (α-EGFR) following SDS-PAGE. (**B**) The protein level of RRM2 was quantified from three independent experiments. (Compared to DMSO, ^*^p < 0.05, ^**^p < 0.01, and ^***^p < 0.001.).

To mimic the *in vivo* afatinib therapeutic conditions, we extended the incubation time of afatinib to 48 h with one replacement of the culture medium containing afatinib at 24 h. Under the prolonged treatment conditions, the protein level of RRM2 was significantly reduced by the treatment of 100 nM and 1 μM of afatinib (Figure 5A, lower panel), while EGFR protein levels were increased upon the treatment with 1 nM to 100 nM afatinib (Figure 5A, upper panel). Therefore, the long-term incubation with afatinib significantly lowers the effective concentration of afatinib against RRM2 in cultured cells. In order to exclude the possibility that inhibition of EGFR by afatinib may cause downregulation of other afatinib targets, we chose the EGFR-null CHO cells [31] to examine the effects of afatinib on the protein levels of RRM1 and RRM2. The long-term incubation of afatinib in CHO cells also leaded to decreasing RRM2 protein levels in a dose-response manner (Figure 5B, lower panel). Like PC-9 cells, RRM1 was relatively resistant to the afatinib treatment in CHO cells (Figure 5B, upper panel). We also found that afatinib also could increase the levels of γ-H2AX in CHO cells in a dose-response manner after 24 h treatment (Figure 5C). However, treatment of afatinib at 1 nM to 1 μM did not affect the cell cycle behavior of CHO cells (Supplementary Figure 6). These results indicate that afatinib can cause the decline of RRM2 protein level and induce DNA damage in cells, which are apparently independent of the EGFR signal pathway. In addition, when the duration of afatinib treatment in PC-9 cells was extended to 72 h with daily replacement of the culture medium containing afatinib, the results (Figure 6) further showed that the long-term afatinib treatment could decrease the protein levels of RRM1 and RRM2 and increase the protein levels of EGFR in a dose-response manner in PC-9 cells. The results together indicate that at the therapeutic concentrations (10–100 nM) afatinib mainly cause DNA damage, G1 arrest in cell cycle and growth inhibition, but not cell death of human lung cancer cells.

### Combined treatment of afatinib and gemcitabine

**Figure 7:**
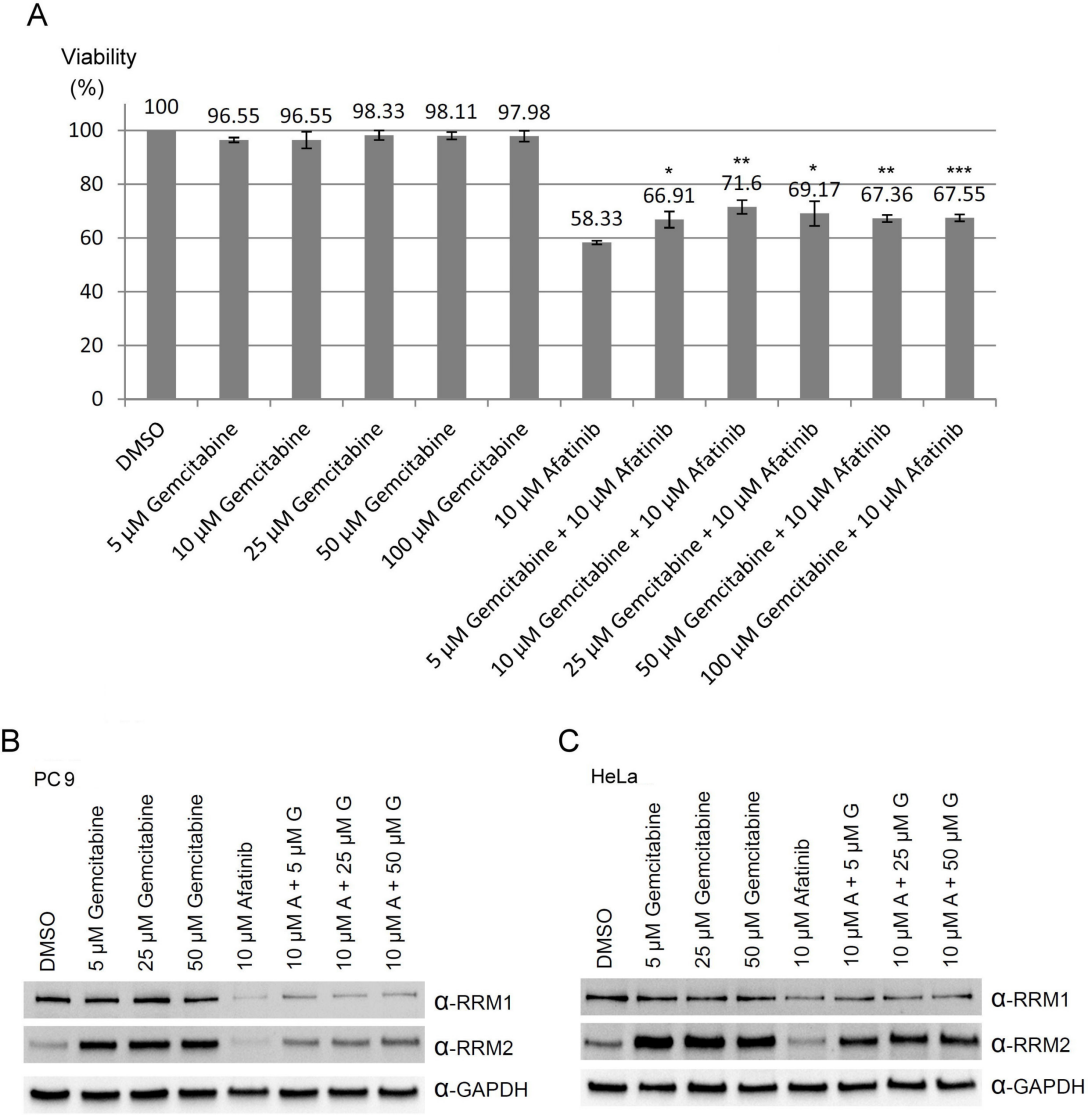
Combination treatments of afatinib and gemcitabine in PC-9 cells (**A**) PC-9 cells were treated with various concentrations of gemcitabine in the absence or presence of 10 μM afatinib in cultured medium containing FBS for 24 h. After the treatment, the cells were washed with TBS for three times, and then cell number counted with the MTT assay. Results were presented as mean of three independent experiments at the same time plus and minus standard deviation. (Compared to 10 μM afatinib, ^*^p < 0.05, ^**^p < 0.005, and ^***^p < 0.001) (**B**) PC-9 cells or (**C**) HeLa cells were treated with various concentrations of gemcitabine in the presence or absence of 10 μM afatinib in cultured medium containing FBS for 24 h. After the treatment, the cells were washed with PBS for three times and then lysed with urea lysis buffer containing 1 μM cysteine. The cell lysate was examined by immunoblotting with anti-RRM1 antibody (α-RRM1) and anti-RRM2 antibody (α-RRM2) following SDS-PAGE.

**Figure 8:**
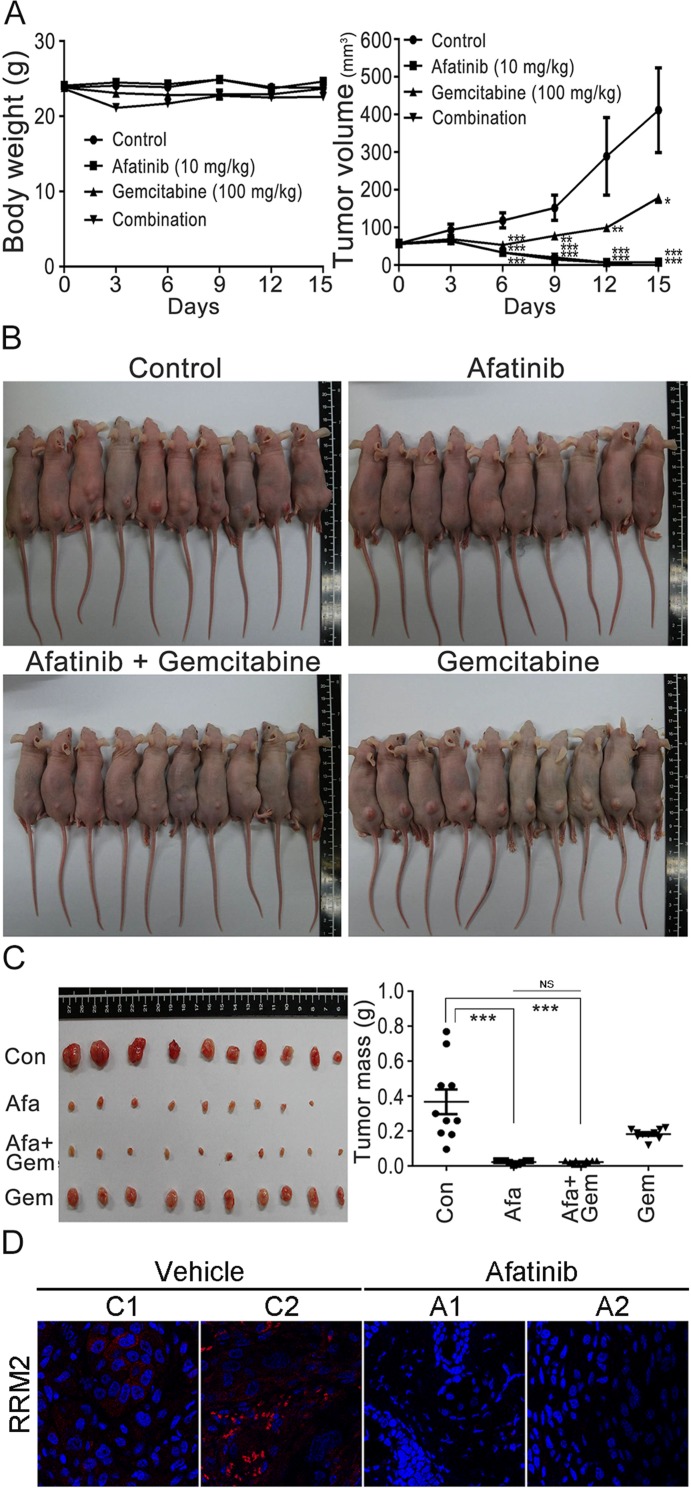
Combination treatments of afatinib and gemcitabine in nude mice (**A**) Effects of afatinib, gemcitabine, or in combination on the tumor growth of PC-9 cells in xenograft mice. After the subcutaneous inoculation of PC-9 cells for 14 days, the nude mice were randomly assigned into four groups for treatment: afatinib group (*n* = 10), gemcitabine group (*n* = 10), afatinib/gemcitabine group (*n* = 10), and control group (*n* = 10). For the treatment, afatinib in sterile water was orally taken with 10 mg/kg daily, and gemcitabine in PBS was orally administrated with 100 mg/kg weekly. Mouse body weights and tumor sizes in appearance were measured and recorded every 3 days. (**B**) Tumors on xenograft mice. After 15-day treatment, the mice in the four groups were aligned to show the tumors on the mouse skin and imaged. (**C**) Tumor lesions and tumor masses. Fifteen days after the treatment, the mice were sacrificed and the tumor lesions were taken out, weighed and photographed. The mouse numbers for each group were 10 (*n* = 10) and one tumor lesion was vanishing after the afatinib treatment in the group. (**D**) Immunofluorescent analysis of RRM2 in the tumor lesions after afatinib treatment using an anti-RRM2 antibody. Nuclei were counterstained with DAPI. Red, RRM2; Blue, nuclei. Amplification, 100X. The tumor masses were statistically calculated and plotted. (^*^p < 0.05, ^**^p < 0.01, and ^***^p < 0.001).

Based on the observations that RNR was one of the afatinib-targeted proteins, we then further examined whether there was a combination effect of afatinib with gemcitabine (a well-known inhibitor of RNR) on the viability, RRM1 and RRM2 protein levels of human lung cancer cells. The results showed that gemcitabine alone had no significant effect on PC-9 cell viability , while gemcitabine treatment could slightly attenuated the cytotoxicity of 10 μM afatinib on PC-9 cells (Figure 7A), in accordance with the results that afatinib and gemcitabine competed for the same binding sites on RRM1 and RRM2 (Figure 2C). Since overexpression of RNR is one of the mechanisms underlying gemcitabine resistance in cancer cells [18], we attempted to study whether the combined treatment of afatinib and gemcitabine could affect the protein levels of RRM1 and RRM2. The results (Figure 7B and 7C) showed that afatinib could decrease the protein levels of RRM1 and RRM2 in PC-9 and HeLa cells, while gemcitabine could increase the protein levels of RRM2 and marginally affect RRM1 in both cells. In the combination treatment, afatinib repressed the protein levels of gemcitabine-induced RRM2 in PC-9 and HeLa cells. The results together indicate that there is no combination effect of afatinib and gemcitabine on RRM2 in human lung cancer cells. To further address whether there was a combination effect of afatinib and gemcitabine on the tumor growth of human lung cancer cells, we examined the efficacy of afatinib, gemcitabine and both in combination on the tumor growth of PC-9 cells in a xenograft mouse model. The results showed that the drug administration alone or in combination had no significant effect on mouse body weights in spite of a slight decline of body weight observed on Day 3 after the first administration of gemcitabine and in combination group (Figure 8A, left panel). Interestingly, afatinib showed a better inhibitory effect on the tumor growth than gemcitabine, and continuously repressed the tumor volumes below the original size (Figure 8A, right panel). Gemcitabine had no combination effect on afatinib-inhibited tumor growth of human lung cancer cells. On Day 15 after the treatment, the tumors on the xenograft mice were shown in Figure 8B. After the mice were euthanized, the tumor lesions were taken out, imaged and weighed. As shown in Figure 8C, afatinib had a greater efficacy on suppressing the tumor masses than gemcitabine and significantly decreases the tumor masses compared to the control group. In the afatinib group, there was one tumor disappeared after the treatment (Figure 8C, left panel). Again, gemcitabine had no combination effect for afatinib to reduce the tumor masses. Immunocytochemical analyses showed that RRM2 protein was almost undetectable in the tumor lesions treated with afatinib in contrast to the control tumor lesions (Figure 8D). Thus, the results together indicate that there is no combination effect of afatinib and gemcitabine on the treatment of lung cancer. One of the explanations for no combination effect is that afatinib and gemcitabine share the same target; RNR. The fact that afatinib had a better efficacy on repressing lung tumor growth than gemcitabine may be due to its additional effects on the inactivation of the EGFR members.

## DISCUSSION

Treatment of afatinib in cellulo caused degradation of RNR proteins resulting in DNA damage in PC-9 cells independent of the EGFR signal pathway since the similar effects can be observed in the EGFR-null CHO cells (Figure 5B and 5C). Treatment of afatinib also caused G1 arrest in PC-9 cells, but not in CHO cells. The data suggest that downregulation of the ribonucleotide reductase and the inhibition of EGFR may cooperatively contribute to the therapeutic effects of afatinib. If afatinib is monospecific to EGFR, cells treated with afatinib would be arrested at G1 [32]. In support of this notion, expression of dominant negative EGFR [33], blockade of EGFR kinase activity by anti-EGFR monoclonal antibody [34, 35] and treatment of non-covalent EGFR kinase inhibitors [36–38] all suppress cell proliferation and induce G1 arrest in a variety of cancer cells with only minimal cytotoxic effects. Additional inhibition of RNR in PC-9 cells by afatinib at the therapeutic concentrations would exert enhanced effects on cell cycle arrest in G1 phase [39]. Therefore, afatinib treatment in CHO cells causes the degradation of RNR proteins resulting in DNA damage, but not G1 arrest possibly because afatinib does not inhibit insulin or fibroblast growth factor receptor. Afatinib treatment at 10 μM for 24 h caused downregulation of EGFR, RRM1, and RRM2 in PC-9 cells (Figures 3 and 5). Afatinib treatment in PC-9 cells for longer time periods caused significant decrease in protein levels of RRM2, and RRM1 at lower concentrations as expected, but not EGFR. Notice that treatment of afatinib at 1 nM-1 μM for 24 h or 48 h caused upregulation of EGFR in PC-9 cells (Figures 3 and 5) indicating afatinib at the therapeutic concentrations does not cause EGFR degradation, however, resulting in upregulation of proteins levels through unknown mechanisms. That inhibition of EGFR by afatinib at 1 nM-1 μM may result in decrease in receptor activation, receptor endocytosis and therefore receptor lysosome degradation [40] in part explains the upregulation of EGFR induced by afatinib.

Tumors with activation EGFR mutations such as deletion mutations in exon 19 and the substitution of leucine with arginine at codon 858 (L858R) are particularly sensitive to EGFR-specific TKIs [41–44]. Afatinib potently inhibits the *in vitro* activity of wild-type and mutant EGFR including L858R and T790M EGFR variants [6], but is ineffective to overcome drug resistance in patients acquiring T790M mutation [45, 46]. It may possibly arise from concentration limitation *in vivo* due to the toxicity associated with inhibition against wild-type EGFR. Interestingly, the combination of afatinib and cetuximab, an EGFR-specific antibody, treatment resulted in extensive tumor shrinkage of erlotinib-resistant tumors harboring the T790M mutation in mice but either agent alone was much less effective [47]. In addition, the combination of afatinib and cetuximab for EGFR-mutant lung cancers with acquired resistance to gefitinib or erlotinib is clinically effective regardless of patients with T790M mutant or not [48]. The data suggest that the advanced NSCLC cells acquiring EGFR mutations still rely on EGFR signaling for survival [47, 48] and additional targets and mechanisms affected by afatinib are involved in addition to the EGFR signaling. For example, afatinib suppressed the transcription of cell proliferation regulating inhibitor of protein phosphatase 2A (CIP2a) leading to activation of protein phosphatase 2A and apoptosis [49]. In addition, afatinib reversed ABCB1-mediated multidrug resistance in ABCB1-overexpressing ovarian cancer cells by inhibiting the efflux function of ABCB1 [25, 50]. Similarly, direct inhibition of ABCG2 ATPase activity and ABCG2-mediated efflux of ABCG2 substrate were also demonstrated [51]. Our data also suggest that additional inhibition of RNR by afatinib underscores the therapeutic effects of afatinib.

Gemcitabine (2’,2’-difluoro deoxycytidine), converted by deoxycytidine kinase to the active diphosphate and triphosphate, inhibits RNR by binding to RRM1 and DNA polymerase, respectively [28, 52]. RNR catalyzing the formation of deoxyribonucleoside diphosphate from ribonucleotides is essential for the DNA synthesis required for DNA damage repair and cell division. The enzyme remains the preferred chemotherapeutic target in cancer therapeutics. Interestingly, afatinib treatment would down-regulate RNR in PC-9 cells resembling the action of gemcitabine. In support of this notion, combined treatment of afatinib and gemcitabine attenuated the toxicity of afatinib treatment (Figure 7A), possibly due to the upregulation of RRM2 induced by gemcitabine treatment [18]. In addition, treatment of 10 μM afatinib in PC-9 cells caused apoptosis and S-phase and G2/M stalling similar to gemcitabine treatment in other non-small cell lung cancer cells [53, 54]. Afatinib appears to compete for the substrate-binding sites in RNR because afatinib is an analog of ATP, and both ADP and gemcitabine decreased the afatinib labeling to ribonucleotide reductase (Figure 2C). Nevertheless, one major difference differentiates these two drugs; afatinib treatment causes downregulation of RNR protein levels while gemcitabine treatment induces upregulation of RNR protein levels in response to RNR inhibition.

Targeted covalent drugs have recently gained more attention, particularly protein kinase inhibitors [55–57]. These drugs target a non-catalytic nucleophile that is unique for each target protein in contrast to the catalytic nucleophile in mechanism-based or suicide inhibitors. Inspired by the satisfactory outcome from the clinical trials of afatinib and ibrutinib [8, 58–61], more targeted covalent drugs are being designed and synthesized. However, most TKIs are only examined among the kinase family members in the determination of inhibitor specificity. Since most kinase inhibitors are targeting the ATP binding site of protein kinases, they might not be selective because other ATP-binding proteins may have substrate binding sites indistinguishable from those in protein kinases [24]. In addition, small-molecule drugs are not always monospecific; approved small-molecule drugs have up to around seven known targets in average [62]. Our TISTA approach using antiserum against covalent drugs can provide an effective approach in unraveling potential targets and off-targets of targeted covalent drugs in the process of drug development. Although afatinib was intended to target EGFR mutants that were resistant to the first-generation TKIs, multiple targets down-regulated by afatinib may render afatinib preventive against drug resistance. It has been suggested that complex pathologies such as cancer, cardiovascular disease and depression resulting from multiple gene defects are more likely treated with multi-target drugs [63, 64]. Cancer cells treated with monospecific drugs often become refractory due to the activation of alternative pathways. Especially, RNR is legitimate targets of cancer therapy since DNA damage resulting from the inhibition of dNTP synthesis would cause cell-cycle arrest and cell death [65].

Importantly, afatinib may serve as a lead compound for RNR inhibitors and structure–activity relationship analysis of this lead compound may result in better therapeutic agents targeting RNR. Although drug repurposing can be facilitated by in silico approaches, *in vitro* assays, *in vivo* experiments and clinical observations [66], our TISTA method is particularly useful finding direct on-targets and off-targets of covalent drugs, a step critical to the success of repurposing.

We wish to summarize the advantage of using antibody recognition in the identification of potential targets of covalent inhibitors. First, the approach permits in cellulo tagging and immunodetection. Second, the antibody not only recognizes the antigen but also other inhibitors with similar structure. Third, the use of secondary antibodies results in increased sensitivity. It is anticipated that TISTA will be extensively applied in target identification of the targeted covalent drugs and some natural products.

## MATERIALS AND METHODS

### Materials

Gemcitabine, propidium iodide, anti-RRM1, anti-RRM2 (Santa Cruz Biotechnology, Inc., Dallas, TX); DMEM, RPMI 1649 medium, fetal bovine serum (HyClone Laboratories, Inc., South Logan, UT); human RRM1 and RRM2 produced from HEK293 cells (OriGene Technologies, Inc., Rockville, MD); anti-EGFR, anti-GAPDH, DNA damage antibody sampler kit (Cell Signaling Technology, Boston, MA); C18 Zip-tip, Amicon Ultra-15 centrifugal filters, anti-phospho-Histone H2AX (Ser 139) (α-γ-H2AX) (Merck Millipore, Darmstadt, Germany); protein A-sepharose fast flow (GE Healthcare, Chicago, IL ); afatinib, canertinib, dacomitinib, neratinib, erlotinib (LC Laboratories, Woburn, MA) were purchased from manufacturers indicated in parentheses. Other chemicals were mostly from Sigma-Aldrich Corporation (St. Louis, MO).

### Cell culture

PC-9 cells were obtained originally from Sigma-Aldrich Corporation. HeLa cells, NIH3T3 cells, COS1 cells, H441 cells, H3225 cells, and H1975 cells were obtained originally from American Type Culture Collection. All cells were cultured in DMEM or RPMI 1640 medium containing 10% fetal bovine serum (FBS) within 5% CO_2_ atmosphere at 37° C.

### Preparation of anti-afatinib antiserum

The antigen of afatinib was prepared by coupling of the cysteine thiolate in ovalbumin (OVA) to the alkene carbon of afatinib under alkaline conditions. Two ml of OVA at 2 mg/ml in PBS was reduced by 50 mM 1,4-dithioerytreitol at 37° C for 1 h. To this solution, 2 ml of 20% trichloroacetic acid was added. The mixture was mixed. Twenty ml ice-cold acetone was added and the mixture was mixed and kept at −20° C overnight. The resulting precipitate following low-speed centrifugation was dissolved in 2 ml 8 M urea, 0.1 M sodium carbonate buffer, pH 9.40, containing 4 mg afatinib and the solution was incubated at 37° C for 4 h. The protein samples were buffer-exchanged into phosphate-buffered saline (PBS) by centrifugal concentration using an Amicon device with a cutoff of 10 kDa (Merck Millipore) and then used for routine subcutaneous immunizations in guinea pigs. Following six biweekly injections, whole blood was collected from the anesthetized animals 10 days after the final injection.

### Afatinib tagging in cellulo

For dose-dependent treatment of afatinib, PC-9 cells were washed with Tris-buffered saline (TBS) two times and refreshed with DMEM containing various concentrations of afatinib for 1 h. For time-dependent treatment of afatinib, PC-9 cells were washed with TBS two times and refreshed with DMEM containing 10 μM afatinib for different periods of time. For pH-dependent treatment of afatinib, PC-9 cells were washed with TBS two times and refreshed with a buffer containing 10 μM afatinib for 1 h (50 mM MES pH 6.20, 50 mM MOPS pH 7.20, or 50 mM bicine pH 8.20 containing 127 mM NaCl). Treatment of 10 μM canertinib, 10 μM dacomitinib, and 10 μM neratinib in DMEM for 1 h were also performed following PC-9 cells washed with TBS two times. The other cancer cell lines (HeLa, H441, H3225, and H1975) were washed with TBS two times and refreshed with DMEM containing 10 μM afatinib for 1 h. After drug treatment, the cells were washed with TBS two times and immediately lysed with the lysis buffer containing 8 M urea, 2% CHAPS, and 1 μM cysteine in 100 mM MOPS, pH 7.20.

### Immunoprecipitation

After treatment with 10 μM afatinib for 1 h, the treated PC-9 cells were lysed with IP lysis buffer (50 mM MOPS, pH 7.20, 100 mM NaCl, 1 mM EDTA, 5% glycerol, and 1% NP-40) containing protease inhibitor cocktails. After centrifugation, the supernatant was added with SDS to a final concentration 0.3% and heated at 65° C for 10 min. The solution was cooled on ice and added with 9× volume of IP lysis buffer to dilute the SDS. Then, the solution was added with anti-afatinib and incubated at 4° C overnight. The antibody was then pulled down by protein A-conjugated resin and washed by IP lysis buffer for three times. Proteins were eluted by SDS sample buffer. For immunoprecipitation of EGFR, RRM1, and RRM2, the supernatant was directly added with primary antibody and incubated at 4° C overnight. The antibody was then pulled down by protein A-sepharose and washed by IP lysis buffer three times. Proteins were then eluted by SDS sample buffer.

### In-gel digestion of proteins separated by SDS-PAGE for MS analysis

After the SDS-PAGE fractionation, the gel band was cut into small pieces and reduced with 1,4-dithioerythreitol (50 mM) at 37° C for 1 h and alkylated with iodoacetamide (100 mM) at room temperature for 1 h. The gel pieces were destained repeatedly with 25 mM ammonium bicarbonate in 50% acetonitrile until became colorless. Gel slices were dehydrated with 100% acetonitrile for 5 min and vacuum-dried for 5 min. In-gel tryptic digestion was carried out at an enzyme-to-substrate ratio of 1/40 at 37° C for 16 h. The tryptic peptides were extracted twice with 50% acetonitrile containing 5% formic acid under moderate sonication for 10 min and dried completely under vacuum. The peptide mixtures were desalted by C18 Zip-tip (Millipore, Bedford, MA) and subjected to downstream MS analysis.

### Mass spectrometry analysis of proteins and data processing

The samples were reconstituted in 9% acetonitrile and 0.1% formic acid to give a volume of 4 μL, and loaded onto a C18 column of 75-μm × 250-mm (BEH130, Waters, Milford, MA, USA). The peptides mixtures were separated by online nanoflow liquid chromatography using nanoAcquity system (Waters) with a linear gradient of 5 to 50% acetonitrile (in 0.1% formic acid) in 95 min, followed by a sharp increase to 85% acetonitrile in 1 min and held for another 15 min at a constant flow rate of 300 nl/min. Peptides were detected in an LTQ-OrbitrapVelos hybrid mass spectrometer (Thermo Scientific, San Jose, CA, USA) using a data-dependent CID Top20 method in positive ionization mode. For each cycle, full-scan MS spectra (m/z 300–2000) were acquired in the Orbitrap at 60,000 resolution (at m/z 400) after accumulation to a target intensity value of 5 × 10^6^ ions in the linear ion trap. The 20 most intense ions with charge states ≥2 were sequentially isolated to a target value of 10,000 ions within a maximum injection time of 100 ms and fragmented in the high-pressure linear ion trap by low-energy CID with normalized collision energy of 35%. The resulting fragment ions were scanned out in the low-pressure ion trap at the normal scan rate and recorded with the secondary electron multipliers. Ion selection threshold was 500 counts for MS/MS, and the selected ions were excluded from further analysis for 30 s. An activation *q* = 0.25 and activation time of 10 ms were used. Standard MS conditions for all experiments were: spray voltage, 2.1 kV; heated capillary temperature, 200° C; predictive automatic gain control (AGC) enabled, and an S-lens RF level of 69%. All MS and MS/MS raw data were processed with Proteome Discoverer version 1.4 (Thermo Scientific), and the peptides were identified from the MS/MS data searched against the Swiss-Prot (540732 sequences entries) database using the Mascot search engine 2.3.02 (Matrix Science, Boston, MA). Up to three missed cleavages were allowed; and mass accuracy of 10 ppm for the parent ion and 0.6 Da for the fragment ions. The significant peptide hits defined as peptide score must be higher than Mascot significance threshold (*p* < 0.05) and therefore considered highly reliable. The false discovery rate (FDR) of the peptides and protein groups was set to 1% for the MS/MS spectra automatically processed by Proteome Discoverer for statistical validation and quantification.

### Afatinib labeling to ribonucleotide reductase

The reaction mixture contained PBS, 2 μg recombinant RRM1 and/or 1μg RRM2 in the presence of 12.5 μM afatinib for 1 h at 37° C. For ADP competition assay, the reaction mixture initially contained 1 μg recombinant RRM1 or RRM2 in the presence of various concentrations of ADP. After incubation for 15 min, the reaction solution was added with 10 μM afatinib. The reaction was further incubated at 37° C for 60 min. For gemcitabine competition assay, the reaction mixture initially contained 1 μg recombinant RRM1 or RRM2 in the presence of 2.5 mM gemcitabine. After incubation for 15 min, the reaction solution was added with 10 μM afatinib. All the reactions were stopped by adding the sample buffer with 1 mM 1,4-dithioerythreitol.

### Flow cytometry

After afatinib treatment, PC-9 cells were trypsinized and collected by centrifugation at 500x g for 5 min. The harvested cells were washed with ice-cold PBS two times and then fixed in 70% ethanol at 4° C for overnight. After removing the supernatant, the cell pellets were washed with PBS. The cell pellets were resuspended in 1 mL DNA staining solution (20 μg/mL propidium iodide and 0.2 mg/mL RNase A) for 30 min at room temperature. The stained cells were analyzed by FACSCalibur Flow Cytometry (BD Biosciences, Sparks, MD) to measure the DNA content.

### MTT assay

The cells were sub-cultured for at least 16 h for attachment, and then treated with the drugs for the designated time period. After the treatment, the MTT assays were performed when the cells reached 60–80% confluence. The cells were treated with 0.5 mg/mL MTT in DMEM without the phenol red for 1 h, and DMSO was added to dissolve the crystals following the careful removal of MTT solution. The DMSO solution was further transferred to an ELISA plate and the absorbance at 560 nm with background subtraction at 670 nm were obtained. All experiments were repeated for three times.

### Assay of ribonucleotide reductase activity

The PC-9 cells were aliquoted initially with the same cell number in the 6-well plate, cultured to about 60% confluence, and then each well was washed with 1 mL TBS, pH 7.40 for three times followed by freezing at −80° C overnight. After thawing, each well was added with 200 μL 100 mM MOPS, pH 7.40 containing 0–100 nM afatinib and incubated at 37° C for 1 h under 60 rpm seesawing. Later, each well was supplied with 200 μL reaction buffer reaching a final concentration of 0.5 mM CDP, 1.5 mM ATP, 5 mM 1,4-dithioerytreitol, 5 mM MgCl_2_, and 50 mM MOPS, pH 7.40 and incubated at 37° C for 1 h under 60 rpm seesawing. After the reaction, the cells were extracted by 400 μL methanol. The supernatant was collected to a new tube after centrifugation at 12,000 g for 30 min. After the supernatant was vacuum-dried, the pellet was reconstituted in 200 μL alkaline phosphatase reaction solution containing 10 mM bicine pH 8.30, 5 mM MgCl_2_, 0.1 mM ZnCl_2_, and 700 mU/mL alkaline phosphatase and incubated at 37° C for 1 h. The reaction was stopped by addition of 600 μL methanol, and then the supernatant was collected to a new tube after centrifugation at 12,000 g for 30 min. The supernatant was vacuum-dried and reconstituted in 50 μL of ultrapure water for C18 column LC-ESI-MS analysis. The conditions of HPLC and Mass spectrometric analyses were described in our previous publication [67].

### Tumor xenograft animal studies

All procedures for animal experimental protocols were approved by the institutional Animal Care and Use Committee (IACUC) of the College of Medicine, National Taiwan University. Six-week old male BALB/c nude mice were maintained under specific pathogen-free conditions. PC-9 cells (1×10^6^ cells resuspended in 100 μL Opti-MEM) were inoculated subcutaneously into the right flank per nude mouse. After 14 days, when tumors grew with the volumes of approximate 56–58 mm^3^ and animals had the weights of approximate 23–24 g, the mice were randomly assigned to four groups: afatinib group (*n* = 10), gemcitabine group (*n* = 10), afatinib + gemcitabine group (*n* = 10), and control group (*n* = 10). Afatinib (10 mg/kg) was administered by oral gavage every day. Intraperitoneal injection was used for gemcitabine for the drug delivery into mice [100 mg/kg in PBS, every week (Day 1 and Day 8)]. Sterile water was administered by oral gavage every day and sterile PBS was given to the mice by intraperitoneal injection every week as control treatment. Body weights and tumor sizes were measured and recorded every 3 days. Tumor volumes were calculated using the following equation: volume (mm^3^) = length × width^2^ × 0.5. After 15-day treatment, the mice were euthanized, and tumor lesions and masses were photographed and weighed. The tumor volumes and masses were statistically calculated and plotted.

### Immunofluorescence microscopy

The tumor sections on slides were treated with 4% paraformaldehyde for fixation and permeabilized using 0.1% Triton-X100 in PBS. Samples were stained with an anti-RRM2 antibody (1:100, LifeSpan BioSciences), and then followed by a secondary Alexa568-conjugated rabbit antibody. Nuclei were counterstained with DAPI. Slides were examined and photographed using a confocal microscope (TCS-SP5, Leica Microsystems).

### Statistics

Statistical comparisons were performed using Student's unpaired, two-tailed *t*-test with results expressed as the standard deviation (S.D.). *P*-values less than 0.05 were considered as statistically significant.

### Other biochemical methods

Biochemical methods and immunological methods such as SDS-PAGE and immunoblotting were essentially the same as described in our previous publication [68].

Proteomic mass spectrometry analysis was carried out at the Core Facilities for Protein Structure Analysis located at Institute of Biological Chemistry, Academia Sinica.

## SUPPLEMENTARY MATERIALS FIGURES AND TABLES



## Abbreviations

NSCLC: non-small-cell lung carcinoma
TKI: tyrosine kinase inhibitor
TISTA: target identification by specific tagging and antibody detection
RNR: ribonucleotide reductase
RRM1: ribonucleotide reductase subunit M1
RRM2: ribonucleotide reductase subunit M2
FBS: fetal bovine serum
OVA: ovalbumin
MS: mass spectrometry
AGC: automatic gain control
FDR: false discovery rate
dCDP: deoxycytidine diphosphate
dC: deoxycytidine
C: cytidine
dNTP: deoxyribonucleoside triphosphate
DMSO: dimethyl sulfoxide.
