# Supplementary material for: Antibody-assisted target identification reveals afatinib, an EGFR covalent inhibitor, down-regulating ribonucleotide reductase
Source: Oncotarget. 2018 Apr 20;9(30):21512–29. doi: 10.18632/oncotarget.25177 (PMC5940374; doi:10.18632/oncotarget.25177)
Supplement: Supplementary file 1 [file oncotarget-09-21512-s001.pdf]

Antibody-assisted target identification reveals Afatinib, an EGFR covalent inhibitor, down-regulating ribonucleotide reductase

SUPPLEMENTARY MATERIALS

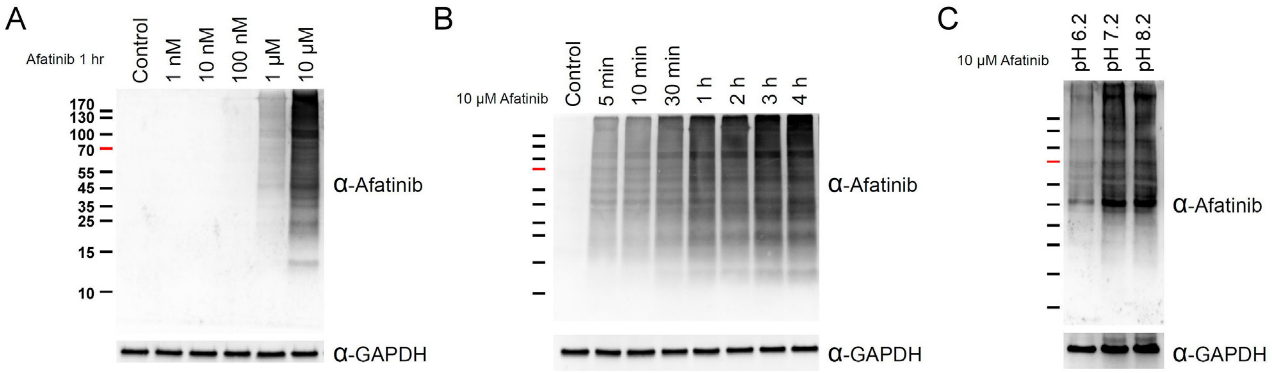

Supplementary Figure 1: Covalent labeling of cellular proteins in cells by afatinib. (**A**) PC-9 cells were treated with afatinib of various concentrations in DMEM without FBS for 1 h. (**B**) PC-9 cells were treated with 10 µM afatinib in DMEM without FBS for 5 min to 4 h. (**C**) HeLa cells were treated with 10 µM afatinib at three different pH values buffers without FBS for 1 h. After treatments of afatinib, the cells were washed with PBS three times and then lysed with urea lysis buffer containing 1 µM cysteine. The cell lysate was examined by immunoblotting with anti-afatinib antiserum (α-Afatinib) following SDS-PAGE. GAPDH was used as an internal control.

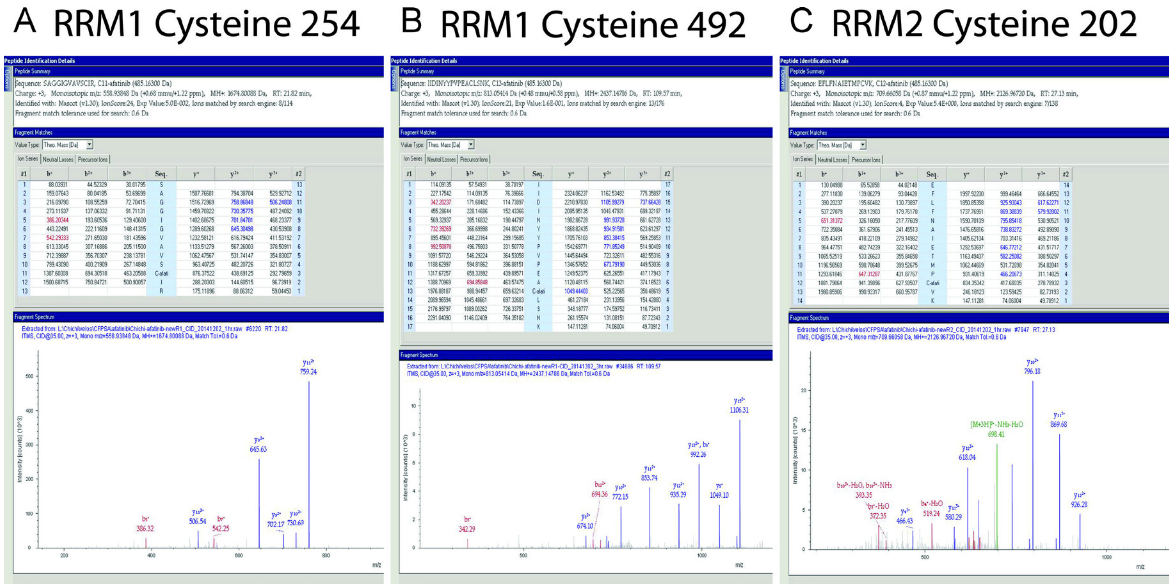

Supplementary Figure 2: Identified modification sites of afatinib in RRM1 and RRM2. Recombinant RRM1 or RRM2 protein purified from HEK293 cells, 1 µg each, was incubated with 12.5 µM afatinib at 37° C for 1 h. After SDS-PAGE and Coomassie blue staining, the gel band was excised and processed to determine the modification sites by MS analysis. Cysteine 254 and cysteine 492 of RRM1 protein and cysteine 202 of RRM2 protein were identified to be tagged with afatinib.

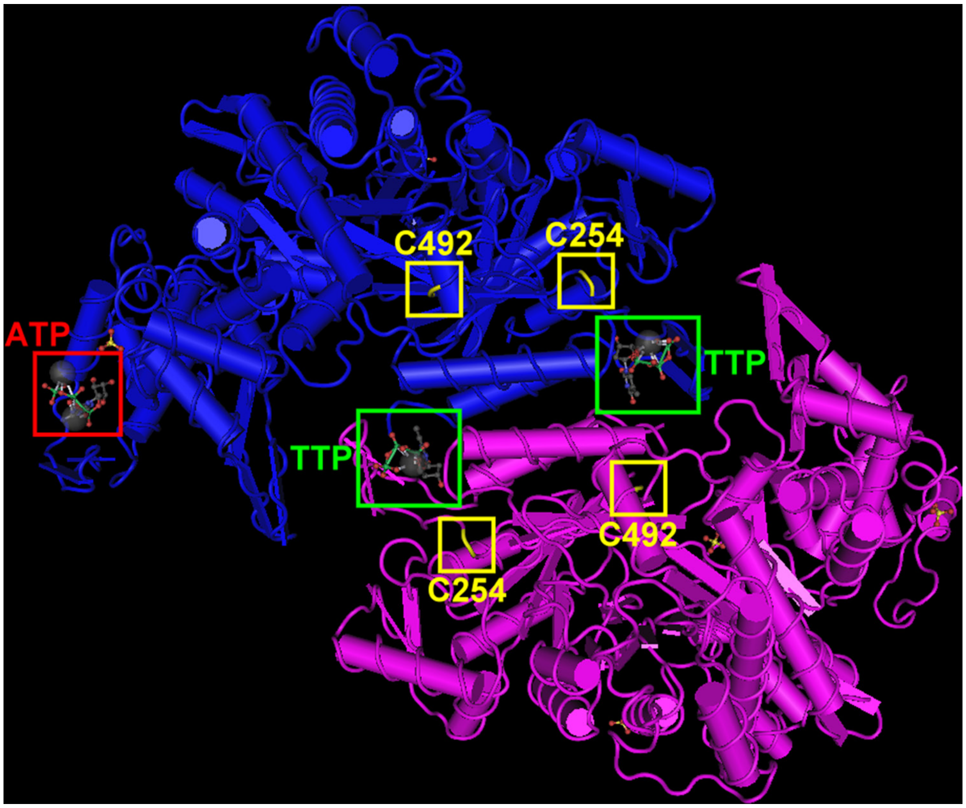

Supplementary Figure 3: Relative positions of Cysteine 254 and Cysteine 492 of human RRM1 to ATP- and TTP-binding sites. The hRRM1-TTP-ATP structure assigned the PDB ID code 3HNE was obtained from Protein Data Bank in Europe. ATP was encircled in red, TTP was encircled in green, and Cysteine 254 (C254) and Cysteine (C492) were encircled in yellow. Fairman JW, Wijerathna SR, Ahmad MF, Xu H, Nakano R, Jha S, Prendergast J, Welin RM, Flodin S, Roos A, Nordlund P, Li Z, Walz T, et al. Structural basis for allosteric regulation of human ribonucleotide reductase by nucleotide-induced oligomerization. Nat Struct Mol Biol. 2011; 18: 316–22. https://doi.org/10.1038/nsmb.2007.

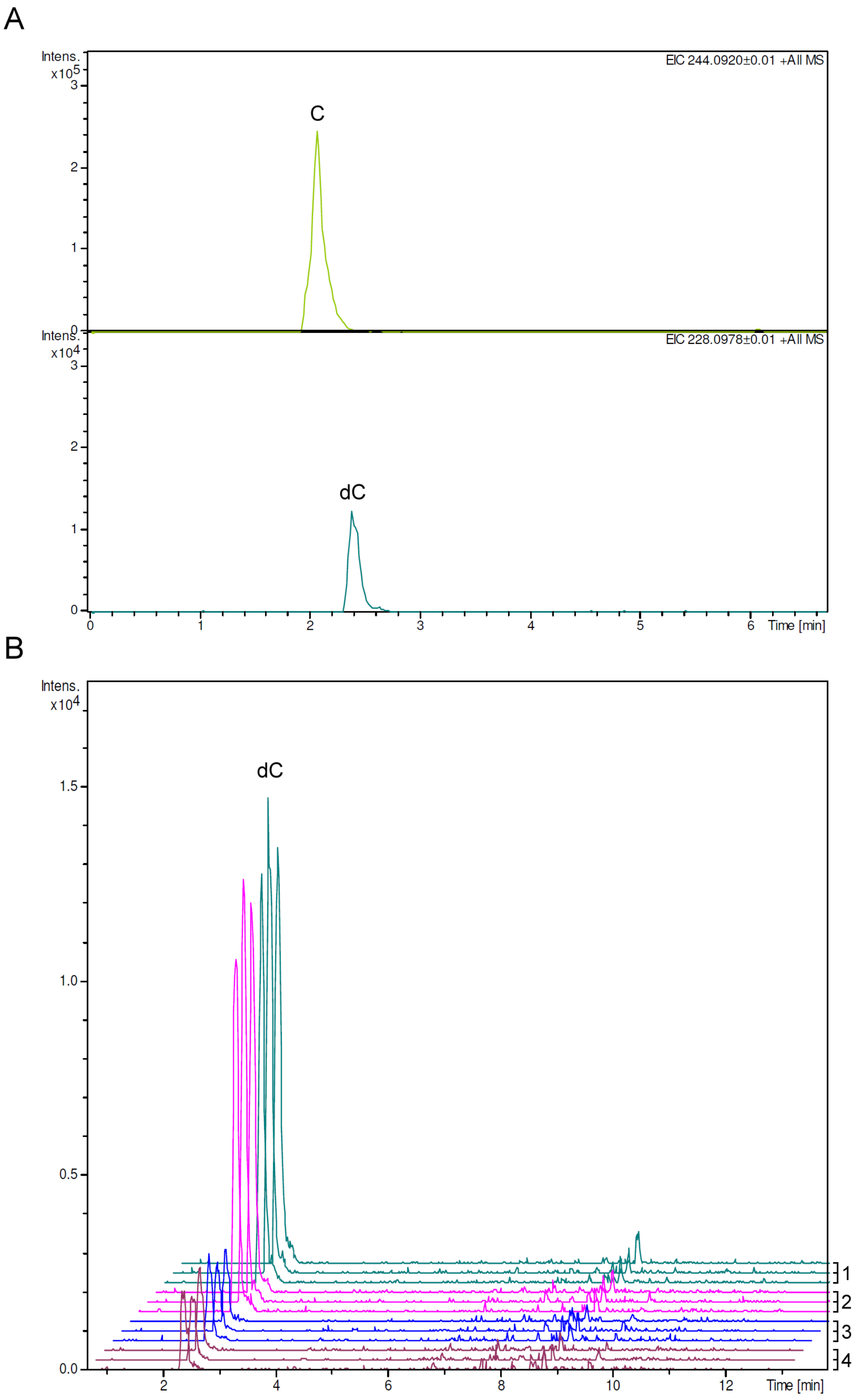

Supplementary Figure 4: Effects of afatinib on ribonucleotide reductase activity. PC-9 cells in rapidly growing phase were frozen at –80° C overnight. After thawing, the lysed cells in the 6-well plate were treated 0-100 nM afatinib and incubated at 37° C for 1 h. Later, each well was supplied with a reaction buffer reaching a final concentration of 0.5 mM CDP, 1.5 mM ATP, 5 mM 1,4-dithioerytreitol, 5 mM $MgCl_2$, and 50 mM MOPS, pH 7.40 and incubated at 37° C for 1 h. After extraction with methanol, the supernatant was vacuum-dried, the pellet was reconstituted in alkaline phosphatase reaction solution containing 10 mM bicine pH 8.30, 5 mM $MgCl_2$, 0.1 mM $ZnCl_2$, and 700 mU/mL alkaline phosphatase and incubated at 37° C for 1 h. The reaction was stopped by addition of 600 µL methanol, and then the supernatant was vacuum-dried and reconstituted in ultrapure water for C18 column LC-ESI-MS analysis. (**A**) LC separation of deoxycytidine (dC) and cytidine (**C**). (**B**) Extracted ion chromatograms and representative mass spectra of deoxycytidine (dC) from all assay samples. 1: no afatinib treatment; 2: 1 nM afatinib treatment; 3: 10 nM afatinib treatment; 4: 100 nM afatinib treatment.

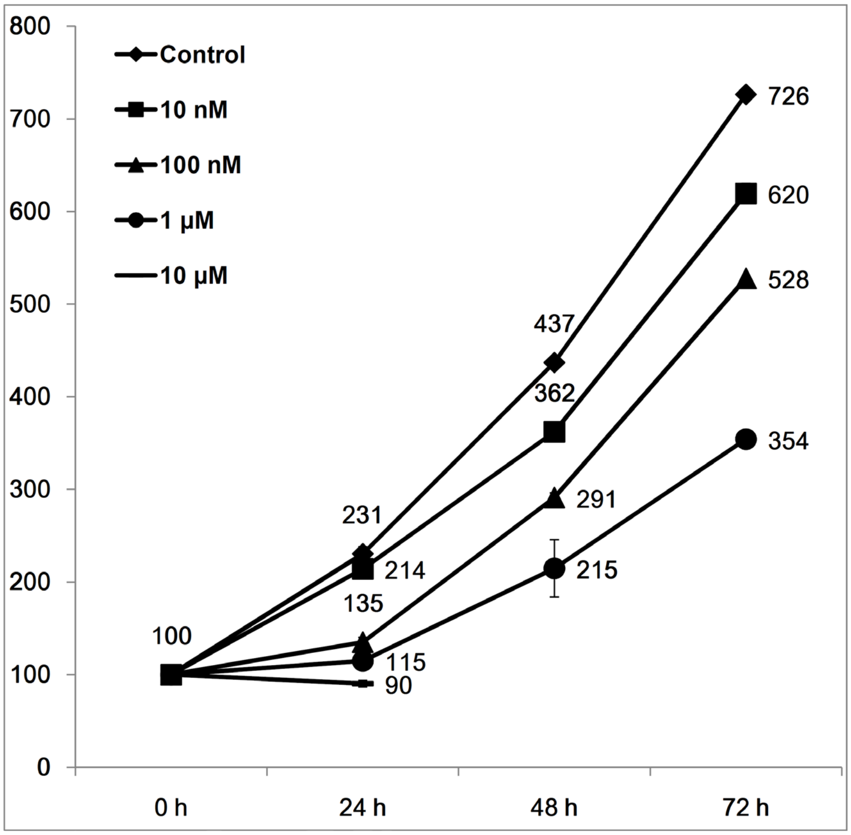

Supplementary Figure 5: Effects of afatinib on PC-9 cell growth. PC-9 cells at a low density were treated with afatinib in cultured medium in the presence of FBS for 72 h, with daily replacement of the culture medium containing afatinib. After drug treatment for 24, 48 and 72 h, the cells were harvested and the cell numbers were counted with the MTT assay.

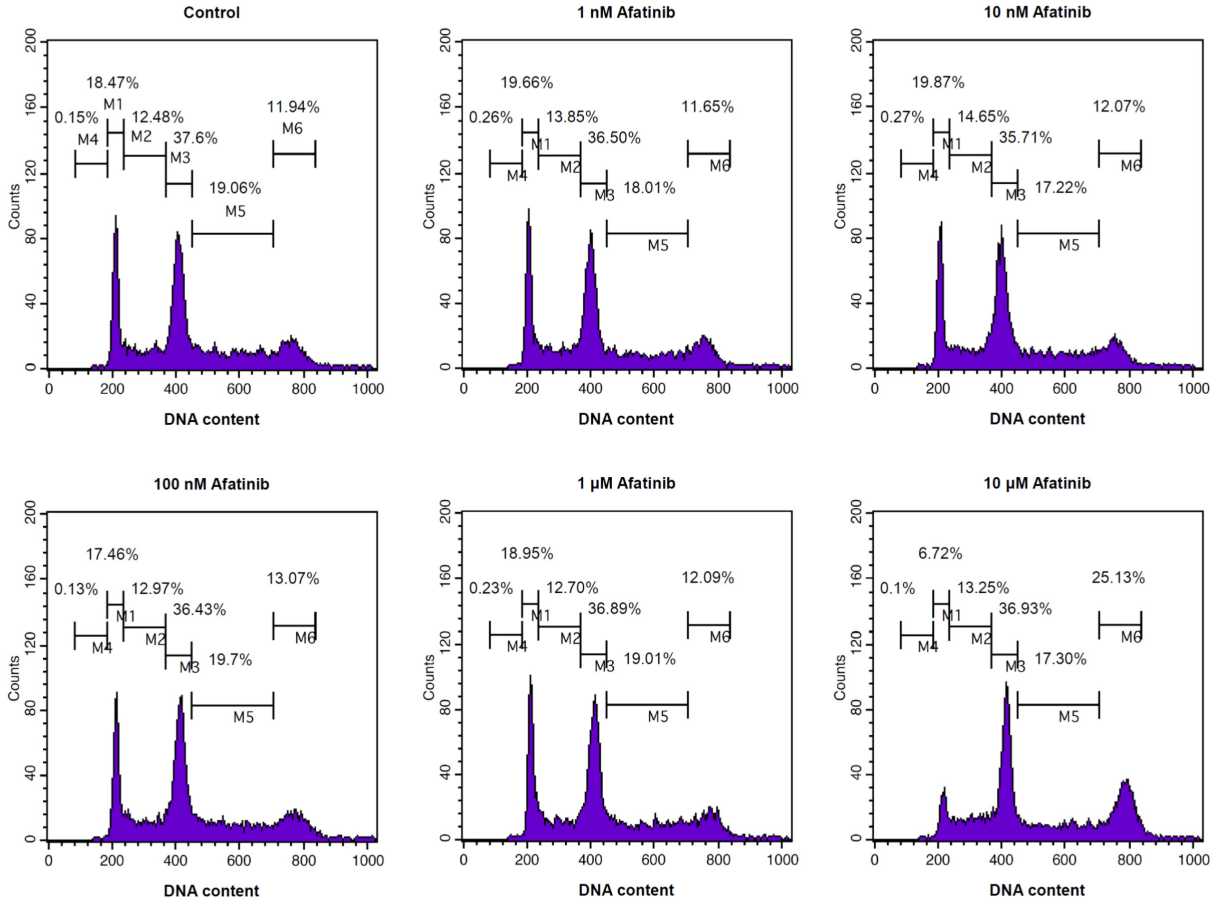

Supplementary Figure 6: Effects of afatinib treatment on cell cycle of CHO cells. CHO cells were treated with 1 nM to 10 μM afatinib in cultured medium containing FBS for 24 h. After propidium iodide staining, the cells were subjected to flow cytometry analysis for cell cycle.
